# Anxiety and Distress Tolerance as Mediators between Complex Posttraumatic Stress Disorder Symptoms and Gambling Severity in Veterans

**DOI:** 10.1007/s10899-025-10449-0

**Published:** 2025-10-31

**Authors:** Glen Dighton, Seb Whiteford, Martyn Quigley, Simon Dymond

**Affiliations:** 1 Present Address: Centre for Military Gambling Research, Swansea University, Swansea, UK; 2 School of Psychology, Swansea University, Swansea, UK; 3 Department of Psychology, Reykjavík University, Reykjavík, Iceland

**Keywords:** Veterans, Gambling, Anxiety, Complex PTSD, Disorders of self-organisation, Distress tolerance

## Abstract

Experiences of gambling-related harm are significant concerns among military veterans, particularly those with posttraumatic stress disorder (PTSD) and complex PTSD (CPTSD). CPTSD, as outlined in the ICD-11, includes disturbances in self-organisation (DSO), encompassing affective dysregulation, negative self-concept, and interpersonal difficulties. While anxiety and distress tolerance (DT) have been implicated in PTSD-related maladaptive behaviours, their roles in the relationship between CPTSD and gambling risk severity remain unclear. This study examines whether anxiety and DT mediate the association between CPTSD symptom clusters (PTSD and DSO) and gambling severity in UK Armed Forces veterans. A cross-sectional study was conducted with UK ex-service personnel (*n* = 346) who completed the International Trauma Questionnaire for CPTSD, the Generalised Anxiety Disorder scale, the Distress Tolerance Scale, and the Problem Gambling Severity Index. Mediation analyses were conducted using bootstrapped regression models. Anxiety was found to be a significant indirect pathway between CPTSD symptoms and gambling risk severity, with a stronger indirect effect observed for DSO symptoms than PTSD-specific symptoms. In contrast, DT did not show a significant indirect pathway, indicating that deficits in DT may not be central to gambling behaviours in veterans with CPTSD. These findings highlight the critical role of anxiety in gambling-related harm among veterans with symptoms of CPTSD, suggesting that interventions targeting anxiety regulation may be beneficial than those targeting distress tolerance in reducing gambling risk severity. Future research should explore additional potential pathways, such as impulsivity and trauma-related dissociation to further clarify associations between CPTSD and gambling severity.

**Supplementary Information:**

The online version contains supplementary material available at 10.1007/s10899-025-10449-0.

## Introduction

Gambling-related harm is a growing public health concern in the UK (Wardle et al., 2024) and worldwide (Abbott, 2020; Tran et al., 2024). It is typified by persistent and recurrent maladaptive patterns of gambling leading to substantial impairment in function, reduced quality of life, and possible diagnosis of gambling disorder (GD; Hodgins et al., 2011). Clinically, GD is highly comorbid with other psychiatric conditions, particularly posttraumatic stress disorder (PTSD) and related affective disorders (Cowlishaw et al., 2014; Ledgerwood & Petry, 2006; Sharman et al., 2019). This comorbid relationship is of particular concern in ex-serving military personnel (i.e., veterans) where exposure to traumatic events is substantially higher than in the general population, and rates of GD and sub-clinical gambling harm/severity are also increased compared to civilians (Dighton et al., 2018; Levy & Tracy, 2018; Etuk et al., 2020). Research suggests that gambling may serve as a maladaptive coping mechanism in veterans with PTSD, offering temporary relief from distressing trauma-related emotions or providing a means of emotional escape (Dighton et al., 2023). However, the specific psychological mechanisms underlying the relationship between PTSD and gambling harm remain underexplored, particularly within the emergent framework of complex PTSD (CPTSD).

CPTSD, introduced in the International Classification of Diseases (ICD-11), extends the traditional PTSD diagnosis by incorporating disturbances in self-organisation (DSO), which include affective dysregulation, negative self-concept, and interpersonal difficulties (Cloitre et al., 2018). The addition of CPTSD aimed to capture the pervasive effects of prolonged or repeated trauma that were poorly represented in earlier diagnostic systems (Reed et al., 2022). Empirical work following its inclusion in the ICD-11 has consistently demonstrated that CPTSD and PTSD are distinct but related trauma-based disorders, with latent class and factor analytic studies across multiple countries identifying two separable symptom profiles (Maercker et al., 2022). Although some debate has focused on the potential overlap between CPTSD and borderline personality disorder (BPD), evidence indicates that the two conditions are more distinct than similar. BPD is characterised by identity instability, impulsivity, and volatile, often intense and short-lived relationships, whereas CPTSD is anchored in trauma and marked by a stable negative self-concept and withdrawal from relationships (Reed et al., 2022; Maercker et al., 2022). This growing consensus supports the ICD-11 decision to classify CPTSD as an independent disorder rather than a severe PTSD subtype. The distinction is particularly relevant in veteran populations, where exposure to cumulative trauma, moral injury, and adverse childhood experiences may contribute to more severe symptomatology and complex patterns of maladaptive coping behaviours (Dighton et al., 2023; Murphy et al., 2021). Specifically, veterans with CPTSD may face additional challenges in emotional regulation and self-concept compared to those with PTSD alone, potentially increasing their vulnerability to gambling-related harms (Dighton et al., 2023). Whilst PTSD has been strongly linked to substance misuse (Brady et al., 2021) and risky decision-making (James et al., 2014; Tull et al., 2016), there is limited research examining whether CPTSD symptoms, particularly those related to self-organisation difficulties, contribute to gambling risk severity and, in turn, an increase in gambling-related harm. Given that CPTSD symptoms extend beyond re-experiencing and avoidance, it is plausible that DSO-related impairments (e.g., difficulties in emotional regulation and self-worth) may drive veterans towards gambling as a means of coping with distress. This raises the question of which cognitive-affective mechanisms best explain the relationship between CPTSD and gambling severity. Therefore, a more comprehensive understanding of the relationship between CPTSD symptoms and risk of gambling harm is needed to inform theoretical models and clinical interventions.

Emotional dysregulation is central to the DSO component of CPTSD, suggesting that difficulties in managing and tolerating distress may play a key role in how trauma symptoms relate to maladaptive coping behaviours, such as gambling. Two potential mechanisms of interest in this relationship are anxiety and distress tolerance (DT). Anxiety has been implicated in the development and maintenance of PTSD symptoms, with heightened arousal and avoidance behaviours potentially increasing engagement in maladaptive coping strategies such as gambling (Vujanovic & Zvolensky, 2018). Anxiety sensitivity, the fear of anxiety-related sensations, has been associated with both PTSD severity and impulsive decision-making in community samples, suggesting a possible mediational pathway between CPTSD and gambling behaviours (Schmidt et al., 2007). In contrast, distress tolerance refers to an individual’s ability to endure heightened emotional arousal without resorting to avoidance or other maladaptive coping strategies, making it another key affective process relevant to trauma-related behaviours (Leyro et al., 2010; Simons & Gaher, 2005). Lower distress tolerance has been associated with both more severe PTSD symptoms in veteran samples (Vujanovic et al., 2011) and increased gambling severity, with individuals who struggle to manage distress more likely to engage in gambling as a form of avoidance in general population samples (Williams et al., 2012).

Recently, we examined the mediational role of anxiety and distress tolerance in the relationship between CPTSD symptoms and alcohol use among UK veterans (Whiteford et al., 2024). In that study, CPTSD was measured using the International Trauma Questionnaire (ITQ; Cloitre et al., 2018), an 18-item instrument consisting of two subscales: ITQ-PTSD and ITQ-DSO. These subscales respectively measure core PTSD symptom clusters, i.e., re-experiencing trauma in the present, avoidance, and a persisting sense of current threat, and disturbances in self-organisation, i.e., affective dysregulation, negative self-concept, and disturbances in relationships. We found that anxiety, but not distress tolerance, mediated the association between CPTSD symptoms and alcohol use severity. Additionally, ITQ-DSO symptomology had a greater relative impact on this association than ITQ-PTSD symptomology. This suggests that anxiety may be a more salient factor than distress tolerance in linking CPTSD to maladaptive behaviours, with DSO-related impairments potentially driving these behaviours more strongly than experiencing PTSD symptoms alone. However, it remains unclear whether similar mediational effects exist in the context of gambling severity.

The present study therefore seeks to extend the findings of Whiteford et al. (2024) to gambling harm by investigating the associations between CPTSD symptom clusters (ITQ-PTSD and ITQ-DSO), anxiety, distress tolerance and gambling risk severity among UK veterans. We hypothesised that the impact of a two-factor model of CPTSD as drawn from subscale scores of the ITQ, that is ITQ-PTSD and ITQ-DSO, on gambling risk severity will be mediated by anxiety but not by distress tolerance.

## Method

### Participants and Procedure

Ex-service personnel (i.e., veterans), of the UK Armed Forces, resident in the UK responded to a larger study which originally examined mental health, wellbeing, and gambling behaviour among UK Armed Forces veterans (Dighton et al., 2023). The present study is, therefore, a secondary data analysis of the larger study. The original study protocol was reviewed and received ethical approval by Wales NHS Research Ethics Committee 6 (REC reference 19/WA/0134) and was conducted in accordance with STROBE guidelines (Vandenbroucke, 2007). Participants provided informed consent; they were provided with information about the study prior to their decision to take part in the original study, and for their data to be used in follow-up studies. Participants were reimbursed with a £10 shopping voucher on completion of the optional questions at the end of the study.

Of the original study’s sample, 364 veterans completed at least one question on each of the measures relevant to this study. For these participants, missing data at the item level ranged from 0 to 1.92%. Little’s (1988) test for Missing Completely at Random (MCAR) was non-significant, (χ2 = 679.14 (638), *p* =.126), indicating that the pattern of missingness did not systematically relate to observed data. Consistent with recommendations that listwise deletion produces unbiased estimates when data are MCAR and the proportion of missingness is small (Graham, 2009) listwise deletion was considered appropriate. This led to the exclusion of 18 participants (4.95% of the total), resulting in a final sample of 346 participants.

The sample for this study is mostly male (92.5%), with White-British ethnicity (97.1%), from England (74.0%), in a relationship (76.0%), in paid employment (65.0%), had attained a qualification post-compulsory education (56.4%), living with family (80.1%), are the owners of their own home (65.9%), are not in receipt of benefits (70.8%), served in the Army (69.1%) and were deployed to 2 or more conflicts (53.8%). The mean age was 51.1 (SD = 12.5). The largest groups in other demographic categories were serving in the Armed Forces for 5–9 years (31.8%), and were discharged 25 or more years ago (33.8%), discharged at the end of their engagement period (41.3%). Further details on demographics can be found in Supplementary Table 1.

### Measures

The *Problem Gambling Severity Index* (PGSI; Ferris & Wynne, 2001) is a 9-item screening tool used to assess the severity of gambling risk over the past year. Items are scored on a 4-point scale from 0 (“Never”) to 3 (“Almost always”), with higher scores indicating increased risk of having experienced more severe, or problematic gambling. Scores on the PGSI are summed to categorise respondents according to their gambling risk severity. Scores of 0 refer to those experiencing no negative consequences with their gambling; scores of 1–2 are referred to as low-risk, with respondents having few negative consequences; scores of 3–7 are referred to as moderate-risk, with few to several clearly negative consequences; scores of 8 or more (up to a maximum of 27) are considered to be experiencing problematic patterns of gambling, that is, gambling that affects multiple different life domains (e.g., financial, social/relationships, work/study, emotional/psychological, cultural, and crime activity; Langham et al., 2015) and a possible loss of control. In this study, the PGSI demonstrated excellent internal consistency, with a Cronbach’s α of 0.95, 95% CIs [0.93, 0.96].

The *International Trauma Questionnaire* (ITQ; Cloitre et al., 2018) is an 18-item instrument designed for identifying experiences of probable PTSD and probable complex PTSD (CPTSD) according to ICD-11 criteria. The ITQ consists of four sections: the first two assess probable PTSD, while the latter two identify probable CPTSD. The initial section addresses three PTSD symptom clusters: re-experiencing in the present, avoidance, and a sense of current threat. The second section evaluates how these symptoms impact three areas of life, focusing on functional impairment due to PTSD. The third section examines symptoms from three DSO (Disturbances in Self-Organisation) clusters: affective dysregulation, negative self-concept, and relationship disturbances. The final section assesses how the symptoms from the third section affect three life domains, emphasising functional impairment from CPTSD. Responses are recorded on a 5-point scale, ranging from 0 (“Not at all”) to 4 (“Extremely”). For respondents to score probable PTSD they require a score of 2 or higher in each PTSD symptom cluster and at least one endorsement in the corresponding functional impairment section. Similarly, for probable CPTSD respondents require a score of 2 or higher in each DSO symptom cluster and at least one endorsement in the corresponding functional impairment section, but in addition must also meet the requirements for probable PTSD. Respondents can be categorised as experiencing either probable PTSD or probable CPTSD, but not both. The ITQ is a screening measure and not a substitute for clinical diagnosis by structured interview, which did not occur as part of data collection. In this study, the ITQ PTSD and ITQ DSO scores demonstrated excellent internal consistency (PTSD: Cronbach’s α = 0.93, 95% CI [0.92, 0.94]; DSO: α = 0.94, 95% CI [0.93, 0.95]).

The *Generalised Anxiety Disorder Assessment* (GAD-7; Spitzer et al., 2006) is a 7-item instrument used to evaluate generalised anxiety disorder. Items are rated on a 4-point scale ranging from 0 (“Not at all”) to 3 (“Nearly every day”), reflecting the frequency of symptoms experienced in the past two weeks. Scores are then totalled, and respondents are categorised as experiencing minimal anxiety (scores of 0–4), mild anxiety (scores of 5–9), moderate anxiety (scores of 10–14), or severe anxiety (scores greater than 15). In this study, the GAD-7 total score showed excellent internal consistency (Cronbach’s α = 0.94, 95% CI [0.92, 0.95]).

The *Distress Tolerance Scale* (DTS; Simons & Gaher, 2005) is a 15-item self-report measure that assesses an individual’s ability to tolerate psychological distress. Responses are given on a 5-point scale from 1 (“Strongly agree”) to 5 (“Strongly disagree”) and these endorsements are totalled. The DTS provides both a total score and four subscale scores: tolerance, appraisal, absorption of attention, and efforts to regulate distress. In this study, the DTS total score exhibited excellent internal consistency (Cronbach’s α = 0.94, 95% CI [0.93, 0.95]), and the subscales showed good internal consistency (Tolerance α = 0.81 [0.77, 0.85], Appraisal α = 0.87 [0.85, 0.89], Absorption α = 0.85 [0.82, 0.88], and Regulation α = 0.82 [0.77, 0.85]).

### Data Analysis

All analyses herein were performed using R version 4.4.0 (R Core Team, 2024). Assumption checks (normality, outliers, and multicollinearity) were conducted; normality was evaluated with reference to conventional guidelines (skewness < 2, kurtosis < 7; West et al., 1995), and multicollinearity was assessed using variance inflation factors (VIF). Descriptive statistics and zero-order correlations were computed for all measures based on the total scores. For the mediation model, the indirect associations (ab pathways) of ITQ PTSD and DSO scores (X’s) on PGSI total score (Y) via GAD-7 and DTS (M’s) were estimated using bootstrapping in the *psych* package in R (Revelle, 2021). The mediation model estimated the associations between X and M (a paths), M and Y controlling for X (b paths), the direct effects of X on Y (c paths), and the total effects (c’ paths, summing direct and indirect effects). With two predictors (X’s) and two mediators (M’s), the ab estimates’ magnitude and direction were compared. Bias-corrected bootstrap procedures with the recommended 5000 resamples generated 95% confidence intervals, which is an established approach for mediation models with medium-sized samples (Hayes, 2012). Indirect effects and comparisons with 95% CIs that excluded zero were considered statistically significant (Preacher & Hayes, 2004).

## Results

### Descriptive Analyses and Correlations

Table 1 shows the zero-order Pearson’s correlations and the descriptive statistics for each scale relevant to this study. The correlations shown are all statistically significant; this remains the case after adjusting for multiple comparisons with Benjamini and Hochberg’s false discovery rate correction (Benjamini & Hochberg, 1995). The ITQ PTSD, ITQ DSO, and GAD-7 scales all had strong positive correlations with each other, with weak positive correlations between these scales and PGSI. Moderately negative correlations were observed between the DTS and the ITQ PTSD, ITQ DSO, and GAD-7 scales, with a weak negative correlation being observed between DTS and PGSI. As a diagnostic check, we examined multicollinearity using variance inflation factors (VIF); all values were below 5, indicating no problematic multicollinearity (Hair et al., 2010; O’Brien, 2007).

**Table 1:** Descriptive statistics and zero-order pearson’s correlations for all total-score measures

	PGSI	ITQ PTSD	ITQ DSO	GAD 7	DTS
PGSI	-				
ITQ PTSD	0.37^***^	-			
ITQ DSO	0.36^***^	0.73^***^	-		
GAD 7	0.40^***^	0.71^***^	0.77^***^	-	
DTS	−0.30^***^	−0.60^***^	−0.65^***^	−0.65^***^	-
Mean	3.07	6.82	7.86	5.39	3.29
SD	5.12	6.1	6.66	5.45	0.99
95% BCa CIs	2.54–3.64	6.19–7.49	7.18–8.61	4.84–5.97	3.18–3.39
Range	0–26	0–24	0–23	0–21	1–5
Skewness	1.76	0.71	0.55	1.01	−0.1
Kurtosis	5.33	2.55	2.12	3.23	2.13

*p* <.001 = ***, *p* <.01 = ** *p* <.05 = *. *PGSI*: Problem Gambling Severity Index; *ITQ*: International Trauma Questionnaire; *PTSD*: Posttraumatic stress disorder; *DSO*: Disturbances in Self-Organisation; *GAD-7*: Generalised Anxiety Disorder scale total score; *DTS*: Distress Tolerance Scale. 95% BCa CIs refers to 95% confidence intervals calculated by the bias-corrected and accelerated bootstrap procedure

Table 2 displays the categorisation of survey respondents in the scale diagnostic categories.

**Table 2:** Number of participants (n) and percentage in each diagnostic category, and scoring range for the ITQ, PGSI, and GAD-7

	Diagnostic Category	*n*	%	Scoring Range
ITQ	“Likely PTSD”	16	1.62	ITQ PTSD: 9–22 ITQ DSO: 5–17
	“Likely CPTSD”	58	16.76	ITQ PTSD: 9–24 ITQ DSO: 9–24
PGSI	“Gamblers not at risk”	204	59.00	0
	“Lower risk gamblers”	37	10.69	1–4
	“Moderate risk gamblers”	47	13.58	5–7
	“Severe risk gamblers”	58	16.76	8–27
GAD-7	“No anxiety”	177	51.16	0–4
	“Mild anxiety”	95	27.46	5–9
	“Moderate anxiety”	42	12.14	10–14
	“Severe anxiety”	32	9.25	15–21

*ITQ* International Trauma Questionnaire, *PTSD* posttraumatic stress disorder, *CPTSD* complex posttraumatic stress disorder, *DSO* disturbances in self-organisation, *PGSI* Problem Gambling Severity Index, *GAD-7* Generalised Anxiety Disorder scale total score

### Mediation Analysis

**Fig. 1:**
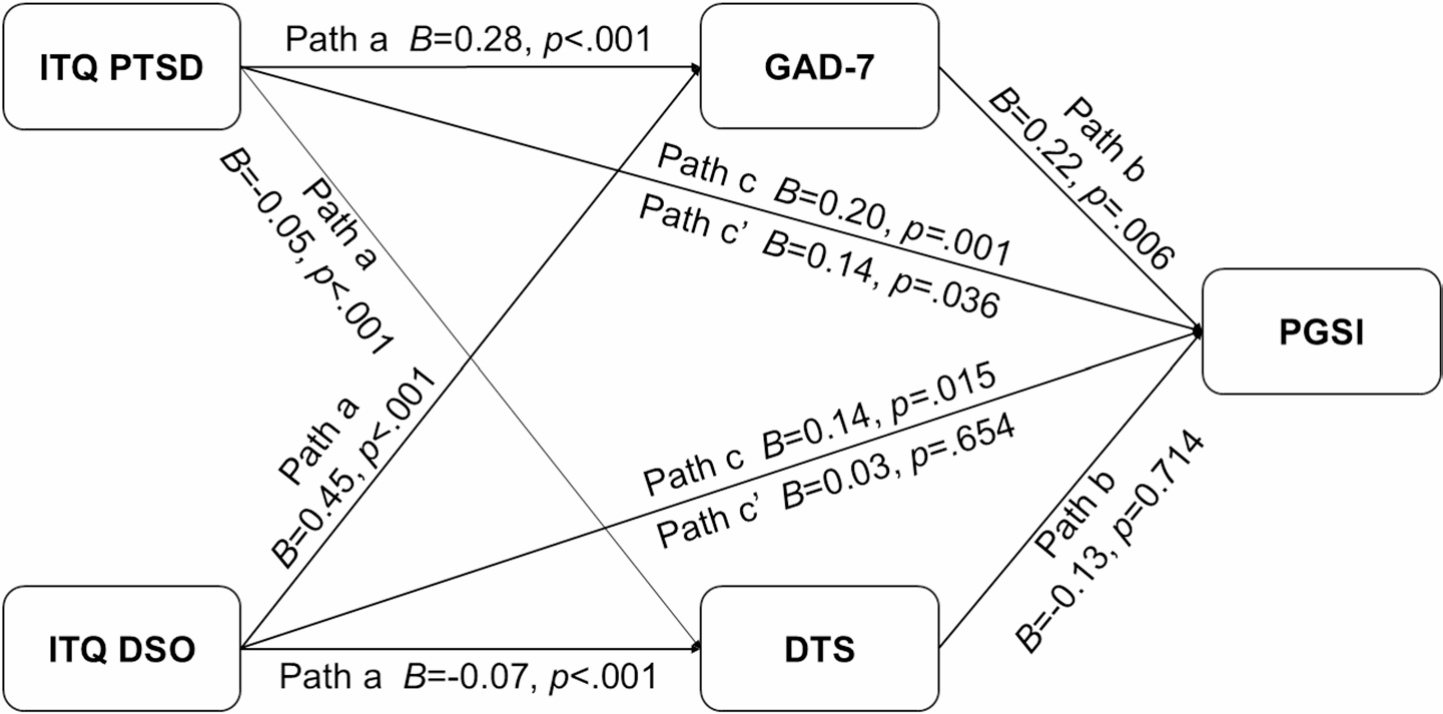
Graphical summary of the mediation analysis pathways. *PGSI*: Problem Gambling Severity Index total score; *DTS*: Distress Tolerance Scale total score; *GAD-7*: Generalised Anxiety Disorder scale total score; *ITQ DSO*: International Trauma Questionnaire Disturbances in Self‐Organisation total score; *ITQ PTSD*: International Trauma Questionnaire PTSD total score

The mediation analysis is summarised graphically in Fig. 1. With direct effects (c’-traditional regression analyses), both GAD-7 total score (*B* = 0.22, *t*(341) = 2.78, *p* =.006) and ITQ PTSD total score (*B* = 0.14, *t*(341) = 2.11, *p* =.036) were statistically significant predictors of PGSI. Whereas ITQ DSO total score (*B* = 0.03, *t*(341) = 0.44, *p* =.661) and DTS total score (*B* = − 0.13, *t*(341) = −0.37, *p* =.714) were not statistically significant predictors of total PGSI score. For the total effects (c) both ITQ PTSD score (*B* = 0.20, *t*(343) = 3.35, *p* =.001), and ITQ DSO score (*B* = 0.14 *t*(343) = 2.45, *p* =.015), were significantly associated with PGSI total scores.

In terms of the “a” pathways (the effect of the independent variables on the mediating variables) both ITQ PTSD score (*B* = 0.28, *t*(343) = 6.59, *p* <.001) and ITQ DSO score (*B* = 0.45, *t*(343) = 11.53, *p* <.001) were statistically significant predictors of GAD-7 and DTS (ITQ PTSD on DTS: *B* = − 0.05, *t*(343) = −4.80, *p* <.001; ITQ DSO on DTS: *B* = − 0.07, *t*(343) = −7.57, *p* <.001). For the “b” pathways (the effect of the mediating variables on the dependent variable, controlling for the independent variables), GAD-7 was a statistically significant predictor of the PGSI total score (*B* = 0.22, *t*(341) = 2.78, *p* =.006), while DTS was not (*B* = −0.13, *t*(341) = −0.37, *p* =.714).

Both ITQ PTSD (*B* = 0.06, 95% CI [0.02, 0.11]) and ITQ DSO scores (*B* = 0.10, 95% CI [0.03, 0.17]) showed indirect effects on PGSI total score via GAD-7 (“ab” pathways), while indirect effects via DTS were not statistically significant (ITQ PTSD: *B* = 0.01, 95% CI [− 0.03, 0.04]; ITQ DSO: *B* = 0.01, 95% CI [− 0.04, 0.06]). All indirect effects were tested using bias-corrected bootstrapped 95% confidence intervals (5,000 resamples). Comparison of the associations suggests a larger indirect effect via GAD-7 for ITQ DSO than ITQ PTSD (mean difference = 0.038, 95% CI [0.002, 0.090]). Other comparisons of indirect effects were not significant. The full model, explaining 18% of the variance in PGSI total scores, was statistically significant (*F*(4,341) = 18.18, *p* <.001, R² = 0.18).

## Discussion

The present study investigated the relationship between CPTSD symptoms and gambling risk severity, examining anxiety and distress tolerance as potential mediators in a sample of UK Armed Forces veterans. Consistent with our hypotheses, anxiety significantly mediated the association between CPTSD symptoms and gambling severity, while distress tolerance did not play a significant mediating role. Specifically, disturbances in self-organisation (DSO) had a stronger indirect effect on gambling severity via anxiety than PTSD-specific symptoms (ITQ-PTSD), reinforcing the notion that affective dysregulation and negative self-concept may be particularly influential in gambling-related behaviours (Cloitre et al., 2018). These findings align with previous work on CPTSD and alcohol misuse (Whiteford et al., 2024), supporting anxiety as a key psychological mechanism linking CPTSD to.

Although prevalence was not the primary focus of this study, 16.8% of the sample met the PGSI threshold for severe gambling risk, substantially higher than UK civilian estimates (0.2%–2.8%; Newall et al., 2024). While the original study’s sample was non-clinical, participants reported elevated PTSD and depression symptoms, both recognised risk factors for gambling (Dighton et al., 2023). Internationally, lifetime prevalence estimates for gambling disorder among military populations range from 2% to 29%, compared to 0.4%–1.6% in general populations (Rayner et al., 2025), indicating that our findings reflect a broader pattern of elevated risk and that our observed rate here is consistent with wider patterns of elevated risk among veterans.

The mediating role of anxiety provides further empirical support for theoretical models suggesting that maladaptive gambling behaviours may function as an emotional regulation strategy in individuals with high anxiety levels (Blaszczynski & Nower, 2009; Goodie & Fortune, 2013). Veterans with CPTSD often experience chronic hyperarousal, excessive worry, and heightened threat perception (Courtois & Ford, 2019), all of which may contribute to increased gambling engagement as a temporary escape from distressing affective states (El-Guebaly et al., 2006). The present findings are also in line with previous research linking anxiety to both PTSD severity (Schmidt et al., 2008; Vujanovic & Zvolensky, 2018) and GD (Broman-Fulks et al., 2014). This suggests that heightened physiological and cognitive awareness of anxious sensations may increase the likelihood of engaging in impulsive gambling behaviours as a means of self-regulation. Given that anxiety-related avoidance is a hallmark of CPTSD, it is plausible that gambling may serve as an avoidance-based coping mechanism, reinforcing continued engagement despite negative consequences (McGinty et al., 2023). Clinically, these findings imply that gambling-focused interventions for veterans may benefit from explicitly addressing trauma-linked anxiety triggers (e.g., through cue exposure and coping-skills training), integrated within evidence-based cognitive-behavioural therapy (CBT) approaches for gambling disorder that include cognitive restructuring and behavioural regulation strategies (Cowlishaw et al., 2014). Such integration may complement trauma-focused treatments like CBT or EMDR, which already emphasise anxiety and affect regulation (Courtois & Ford, 2019).

In contrast, distress tolerance did not significantly mediate the relationship between CPTSD symptoms and gambling severity, which is consistent with (Whiteford et al., 2024), who found no significant indirect effect of distress tolerance on alcohol misuse. While distress tolerance deficits have been linked to various addictive behaviours (Leyro et al., 2010), the present findings suggest that distress tolerance may not play a central role in gambling-related problems among veterans with CPTSD. One possible explanation is that veterans with CPTSD do not necessarily gamble in response to distress tolerance difficulties but rather as a means of managing chronic anxiety. This interpretation aligns with research indicating that distress tolerance is more strongly linked to substance use than to gambling behaviours, as substances may offer an almost immediate relief from distress, whereas gambling may not provide the same level of immediate physiological relief (Dickerson & O’Connor, 2006; Williams et al., 2012; Wood & Griffiths, 2007). Further research is needed to determine whether distress tolerance influences other aspects of gambling behaviour, such as persistence in play or risk-taking tendencies.

In terms of future research directions, it would be useful to investigate alternative mediators, such as impulsivity, experiential avoidance, and trauma-related dissociation, to refine understanding of the psychological mechanisms linking CPTSD and gambling risk severity. Impulsivity, often heightened in trauma-exposed individuals (Billieux et al., 2012), may exacerbate gambling behaviours as a means of immediate emotional relief, particularly given CPTSD-related self-regulation deficits. Similarly, experiential avoidance, suppressing or escaping distressing thoughts, may drive veterans to gamble as a maladaptive coping strategy, reinforcing this as a compulsively driven behaviour (Riley, 2014). Trauma-related dissociation, which impairs reality testing and emotional processing, could further contribute to gambling problems by fostering detachment from financial and social consequences (Petry et al., 2005). Future studies that combine longitudinal methods and clinical outcome data will be essential for evaluating whether anxiety-focused intervention components reduce gambling harm among trauma-affected veterans.

### Limitations

While this study provides novel insights into the psychological mechanisms linking CPTSD symptoms to gambling risk severity in veterans, some limitations should be acknowledged. The cross-sectional design prevents causal inferences, and self-report measures may be influenced by recall bias or social desirability effects. The application of mediational analyses on cross-sectional data has also been debated (O’Laughlin et al., 2018; Rohrer et al., 2022). Whilst we cannot assume that these results would necessarily be replicated with longitudinal analyses, such cross-sectional mediational analyses are commonly used in studies focussing on addiction in veteran populations (Vujanovic et al., 2022; Whiteford et al., 2024) and have merit. Moreover, the bootstrapped regression models employed in the current study are considered more robust than the causal steps approach (Baron & Kenny, 1986) commonly utilised throughout cross-sectional mediational analysis literature (Cain et al., 2018), due to their direct testing of the indirect effects of the model using bias-corrected bootstrapping procedures (Hayes, 2012; Preacher & Hayes, 2004, 2008).

Women comprised 7.5% of the sample, consistent with UK Armed Forces demographics (approx. 8–11%; UK Ministry of Defence, 2024). Excluding women would reduce representativeness and overlook an under-researched subgroup; their inclusion provides essential, albeit limited, insight into the wider veteran population. Hence, these findings should be interpreted as indicative of associations rather than causation; they suggest that anxiety may represent a potential pathway linking CPTSD and gambling severity, which requires confirmation in longitudinal designs. Finally, the sample was predominantly male (92.5%) and White-British (97.1%), reflecting UK veteran demographics but limiting generalisability to more diverse groups.

## Conclusion

This study provides the first empirical evidence that anxiety, but not distress tolerance, mediates the relationship between CPTSD symptoms and gambling risk severity in UK military veterans. The findings highlight the potential importance of affective dysregulation and anxiety in the maintenance of gambling-related harm, suggesting that anxiety reduction strategies may be beneficial for mitigating gambling behaviours among veterans with CPTSD. These results contribute to existing literature by broadening the understanding of CPTSD-related gambling behaviours and reinforcing the importance of targeting anxiety in therapeutic interventions. Future research should explore alternative mediators, such as impulsivity, experiential avoidance, and trauma-related dissociation, to further refine our understanding of the cognitive-affective mechanisms underlying gambling in trauma-exposed populations. Overall, integrating anxiety regulation and emotion-focused CBT techniques within veteran-specific treatment pathways may represent a promising approach to reducing gambling-related harm in those veterans experiencing CPTSD.

## Supplementary Information

Below is the link to the electronic supplementary material.

Supplementary Material 1 (DOCX 18.4 KB)

## Data Availability

The data that support the findings of this study are available from the corresponding author upon reasonable request. The data are not publicly available due to privacy or ethical restrictions.
