# Supplementary material for: Anxiety and Distress Tolerance as Mediators between Complex Posttraumatic Stress Disorder Symptoms and Gambling Severity in Veterans
Source: J Gambl Stud. 2025 Oct 31;42(1):425–38. doi: 10.1007/s10899-025-10449-0 (PMC13009033; doi:10.1007/s10899-025-10449-0)
Supplement: Supplementary file 1 — Supplementary Material 1 (DOCX 18.4 KB) [file 10899_2025_10449_MOESM1_ESM.docx]

**Supplementary Table 1**: Socio-demographics and military demographics of veterans (N=346) included in mediational analyses.

|  | Veterans (N=346) | |
| --- | --- | --- |
|  | n | % |
| Age Group |  |  |
| 19-29 | 15 | 4.34 |
| 30-39 | 54 | 15.61 |
| 40-49 | 72 | 20.81 |
| 50-59 | 109 | 31.50 |
| 60-69 | 77 | 22.25 |
| 70+ | 19 | 5.49 |
| Gender |  |  |
| Male | 320 | 92.49 |
| Female | 25 | 7.23 |
| Ethnicity |  |  |
| White-British | 336 | 97.11 |
| Not White-British | 10 | 2.89 |
| Country of Residence |  |  |
| England | 256 | 73.99 |
| Wales | 37 | 10.69 |
| Scotland | 36 | 10.40 |
| Northern Ireland | 10 | 2.89 |
| Outside the UK | 7 | 2.02 |
| Relationship Status^a^ |  |  |
| In a Relationship | 263 | 76.01 |
| Not in a Relationship | 83 | 23.99 |
| Employment Status^b^ |  |  |
| Employed | 225 | 65.03 |
| Not Currently Employed | 121 | 34.97 |
| Highest Qualification^c^ |  |  |
| No Formal Qualifications | 24 | 6.59 |
| Compulsory Education | 127 | 34.89 |
| Further Education | 195 | 56.36 |
| Household Arrangement |  |  |
| Live Alone | 69 | 19.94 |
| Live with Family/Other | 277 | 80.06 |
| Accommodation Type |  |  |
| Homeowner | 228 | 65.90 |
| Renting or Supported Accommodation | 118 | 34.10 |
| Benefits Status |  |  |
| Not Receiving Benefits | 245 | 70.81 |
| In Receipt of Benefits | 101 | 29.19 |
| Armed Forces Branch |  |  |
| Army | 239 | 69.08 |
| Royal Navy | 58 | 16.76 |
| Royal Air Force | 49 | 14.16 |
| Length of Service |  |  |
| 0-4 Years (Early Leavers) | 51 | 14.74 |
| 5-9 Years | 110 | 31.79 |
| 10-19 Years | 84 | 24.28 |
| 20+ Years | 101 | 29.19 |
| Deployments |  |  |
| Not Deployed | 46 | 13.29 |
| Deployed to One Conflict | 114 | 32.95 |
| Deployed to Multiple Conflicts | 186 | 53.76 |
| Years Since Discharge |  |  |
| 0-8 Years | 85 | 24.57 |
| 9-13 Years | 57 | 16.47 |
| 14-24 Years | 87 | 25.14 |
| 25+ Years | 117 | 33.82 |
| Discharge Type^d^ |  |  |
| End of Engagement | 143 | 41.33 |
| At Own Request (PVR) | 114 | 32.95 |
| Medical | 38 | 10.98 |
| Other | 51 | 14.74 |

Note: ^a^ In a Relationship refers to veterans that are In a Relationship, Co-habiting with a partner, Married, or Married (second or more marriage); Not in a Relationship refers to veterans that are Single, Separated, Divorced, or Widowed. ^b^ Employed refers to veterans that are In Paid Employment; Not Currently Employed refers to veterans that are Unemployed/Actively Seeking Employment, Retired, Not Working Due to Long Term Illness, Unemployed/Not Actively Seeking Employment, In Training/Education, or Looking After Home/Family. ^c^ Compulsory Education refers to veterans that have achieved Level 1 or Level 2 qualifications as required by attendance to compulsory education; Further Education refers to veterans that have achieved optional Level 3 or higher qualifications. ^d^ Discharge Type Other includes Administrative Discharge, Redundancy, Compulsory Discharge, Tempramental Unsuitability.
